# Functional toner for office laser printer and its application for printing of paper-based superwettable patterns and devices

**DOI:** 10.1038/s41598-023-39729-8

**Published:** 2023-08-03

**Authors:** Yanhua Liu, Xingfei Liu, Juanning Chen, Zhuanli Zhang, Libang Feng

**Affiliations:** School of Materials Science and Engineering, Lanzhou Jiaotong University, Lanzhou, 730070 China

**Keywords:** Materials for devices, Nanoscale materials

## Abstract

Laserjet printing is a kind of facile and digital do-it-yourself strategies, which is of importance to fabricate inexpensive paper-based microfluidic devices. However, the printed hydrophobic barrier is not hydrophobic enough due to the weak hydrophobicity and requires subsequent heating, which can lead to the pyrolysis of cellulose in the paper and influence the detection results. Here, for the first time, we report a kind of functional toner including toner and polydopamine (PDA) nanocapsules which contains oleic acid modified ferric tetroxide (OA-Fe_3_O_4_) and octadecylamine (ODA), which is suitable for printing with desired shapes and sizes to lead to formation of superhydrophobic barriers. Moreover, patterns printed with functional toner have good stability, including resistance to moisture, ultraviolet (UV) and bending. Finally, a proof-of-concept of metal and nitrite ions testing is demonstrated using colorimetric analysis, and the results show that the printed devices successfully perform instant detection of ions. The developed functional toner offers easy fabrication, cost-effectiveness and mass production of paper-based devices. In general, this strategy provides a new idea and technical support for the rapid prototyping of microfluidic paper-based analytical devices (μPADs) using laserjet printing.

## Introduction

The potential to transform paper into smart microfluidic chips, just as microfluidic paper-based analytical devices (μPADs)^1–3^, has given way to a number of point-of-care detections, involving environmental monitoring^4,5^, food safety analysis^6,7^, biochemical analysis and clinical diagnosis^8–11^. According to the concept of μPADs was reintroduced by the Whiteside’s group in 2007^12^, fabrication methods still apply a similar approach to that of paraffin-patterned paper, that is, creating a defined hydrophobic barrier surrounding a hydrophilic channel of paper. To prepare superwettable μPADs, present methods have invoked wax printing^13^, screen printing^14^, inkjet etching and printing^15–20^, flexography printing^21^, photolithography^22^, vapor phase deposition^23,24^, laser treatment^25–28^, hand-held or automated tools^29,30^, cutting^31^ etc.

Some of the above-mentioned methods, such as lithographic processes and vapor phase deposition, provide high-resolution and complex patterns, but require expensive equipment and complex steps, making it difficult to meet commercial requirements^32^. The facile manual methods, such as cutting and hand-made fabrication, offer low-cost and simple manufacturing steps, but these methods are not easy to scale up^30^. Among the mentioned technologies, low-cost printing technologies, such as wax printing and inkjet printing, are promising fast prototypes. However, wax printers are not usually available since the use of materials such as wax causes low resolution and inhomogeneity, inkjet printers also require modified inks for production of PADs, which poses several challenges for preparing different inks for each printing^33^.

As a facile, rapid, ultra-cheap, and do-it-yourself fabrication technology, laserjet printing holds great promise in fabricating μPADs^26–28,34,35^. Rajesh Ghosh et al. report, for the first time, a simple, novel and facile method to pattern microfluidic paper-based analytical devices using a laser printer (LP-μPAD)^26^. Ng et al. also reported the fabrication of μPADs using the most widely available type of printer, namely toner laser printers^27^. Although laser printing with commercial toners provides easy and fast prototyping and significantly reduces manufacturing costs and complexity, the high-temperature heating of filter paper in previous works has caused the pyrolysis of cellulose in paper. The pyrolysis results in the formation of aldehydes that can interfere with molecular assays involving redox reactions, which may affect detection effectiveness. In order to overcome this problem, they have confirmed that the removal of the aldehyde can be readily achieved by washing the μPADs with aqueous bleach, but the cleaning process can cause wrinkling or breakage of paper substrate. Hence, the main disadvantages of toner used in laser printer are low water resistance and high-temperature heating required (165–200 °C). All these challenges necessitate the need for preparing functional toner which can be used to print superhydrophobic patterns on paper with additional heating at lower temperature.

Herein, for the first time, we reported a kind of functional toner prepared by mixing polydopamine nanocapsules with commercial toner. The functional toner can be loaded into toner cartridges and used in the printing of various superwettable patterns on paper by the laser printer. The printed pattern is very stable and can resistant to UV irradiation, humidity and mechanical bending. Because of the widest available laser printer, the prepared functional toner can also be used to manufacture PADs at any requirement location. Further, we have used the functional toner-based paper devices for the rapid (< 20 s) and qualitative detection of metal ions, as well as the quantitative analysis of nitrite ion. We believe that the reported technique, which converts ordinary toner into functional toner, can provide an idea for paper-based devices fabricated by laser printing.

## Materials and methods

### Materials

Dopamine was purchased from Acros Organic. Octadecylamine (ODA) and tris (hydroxymethyl) aminomethane (Tris) were obtained from Shanghai Chemical Regent Co., Ltd (China). Iron (III) chloride hexahydrate (FeCl_3_·6H_2_O, 98%), iron (II) chloride tetrahydrate (FeCl_2_·4H_2_O, 98%), ammonium hydroxide (NH_3_·H_2_O, 28–30%), anhydrous ethanol (AR), sodium hydroxide (NaOH, AR), dimethylglyoxime (DMG) and nickel chloride hexahydrate (NiCl_2_·6H_2_O, 98%) were purchased from Sinopharm Chemical Reagent Co., Ltd (China). Oleic acid (OA, 99%) was purchased from J&K Chemical Ltd. The p-aminobenzene sulfonic acid (C_6_H_7_NO_3_S, AR) and alpha naphthylamine (α-C_10_H_9_N, AR) were obtained from Macklin Biochemical Co., Ltd. Sodium bicarbonate (NaHCO_3_, AR) and acetic acid (Ac, AR) were produced from Shanghai Aladdin Biochemical Technology Co., Ltd. All the chemical agents were used as obtained.

### Preparation of functional toner

As previously reported, Fe_3_O_4_ magnetic nanoparticles were synthesized using co-precipitation methods and then surface modifications with oleic acid^36^. Subsequently, ODA and OA-modified Fe_3_O_4_ were added in deionized water by supersonic stirring to form emulsion. The formed emulsion was then dispersed in tris–HCl buffer (pH = 8.5) with 0.5 mg/mL dopamine under mechanical stirring for 24 h at room temperature to form PDA@ODA-OA-Fe_3_O_4_ nanocapsules^37^. The formation mechanism of PDA@ODA-OA-Fe_3_O_4_ nanocapsules is displayed in Supplementary Fig. S1. Finally, lyophilized PDA@ODA-OA-Fe_3_O_4_ nanocapsules were mixed uniformly with toner to obtain homogeneous functional toner. An empty cartridge was refilled with the functional toner and the cartridge was inserted into the laser printer before the printing process was carried out on paper substrate.

### Printing of superwettable patterns by laser printer

Microsoft PowerPoint 2016 software was utilized to design the patterns such as uniform pattern and grey pattern and then the designed patterns were printed on paper using laser printer (HP LaserJet 1020 plus) containing the as-prepared functional toner. The printed samples were evaluated using water contact angle (WCA) tests to optimize the ratio of PDA nanocapsule and commercial toner.

### Evaluation of the durability of printed patterns

The printed superhydrophobic patterns on paper substrate were exposed to severe physical insults (i.e., ultraviolet irradiation, high-humidity atmosphere and bending cycles) to evaluate the durability of its superhydrophobicity. Regular measurements of static WCAs and SAs were performed to monitor the evolution of coating properties along with ultraviolet irradiation, high-humidity atmosphere and bending cycles. The UV irradiation test was performed using ultraviolet light of 365 nm in 24 h with repeated 10 days. The humidity resistance was tested in standard curing box with humidity of 95% at 20 °C in 24 h and the test was repeated 10 days. The bending test was performed by hand with repeated bending of 100 cycles.

### Fabrication of μPADs using laser printer with functional toner

The desired μPADs were designed using Microsoft PowerPoint 2016 software and the hydrophobic barrier was printed on filter paper using the laser printer. All the designs were printed with default settings. The printed paper was then heated uniformly in an oven 30 min at 80 °C to impregnate the paper with ODA (Melting point = 50–60 °C) capsuled in PDA@ODA-OA-Fe_3_O_4_ nanocapsules to from hydrophobic barriers, and then the printed μPADs were ready for colorimetric detection.

### Characterizations

The morphologies of pristine and printed filter papers were observed by scanning electron microscopy (SEM, JSM-6701F, Japan), and TEM images were obtained on a FEI Tecnai G2 F30 transmission electronic microscope. Fourier transform infrared (FT-IR) spectroscopy was performed to investigate the characteristics of the specimens with KBr pellets on a Nicolet is10 instrument (Thermo Scientific). Water-droplet contact angle (CA) values were acquired using a DSA-100 optical contact-angle meter (Kruss Co., Ltd., Germany) at ambient temperature. A 10 μL amount of deionized water, juice, coffee and milk was dropped onto the samples using an automatic dispense controller, respectively and the CAs were determined automatically using the Laplace-Young fitting algorithm. Average CA values were obtained by measuring the sample at five different positions, and all the images were captured with a digital camera (Sony, Ltd., Japan).

## Results and discussion

### Scheme of preparation of functional toner and printing of superwettable patterns

**Figure 1:**
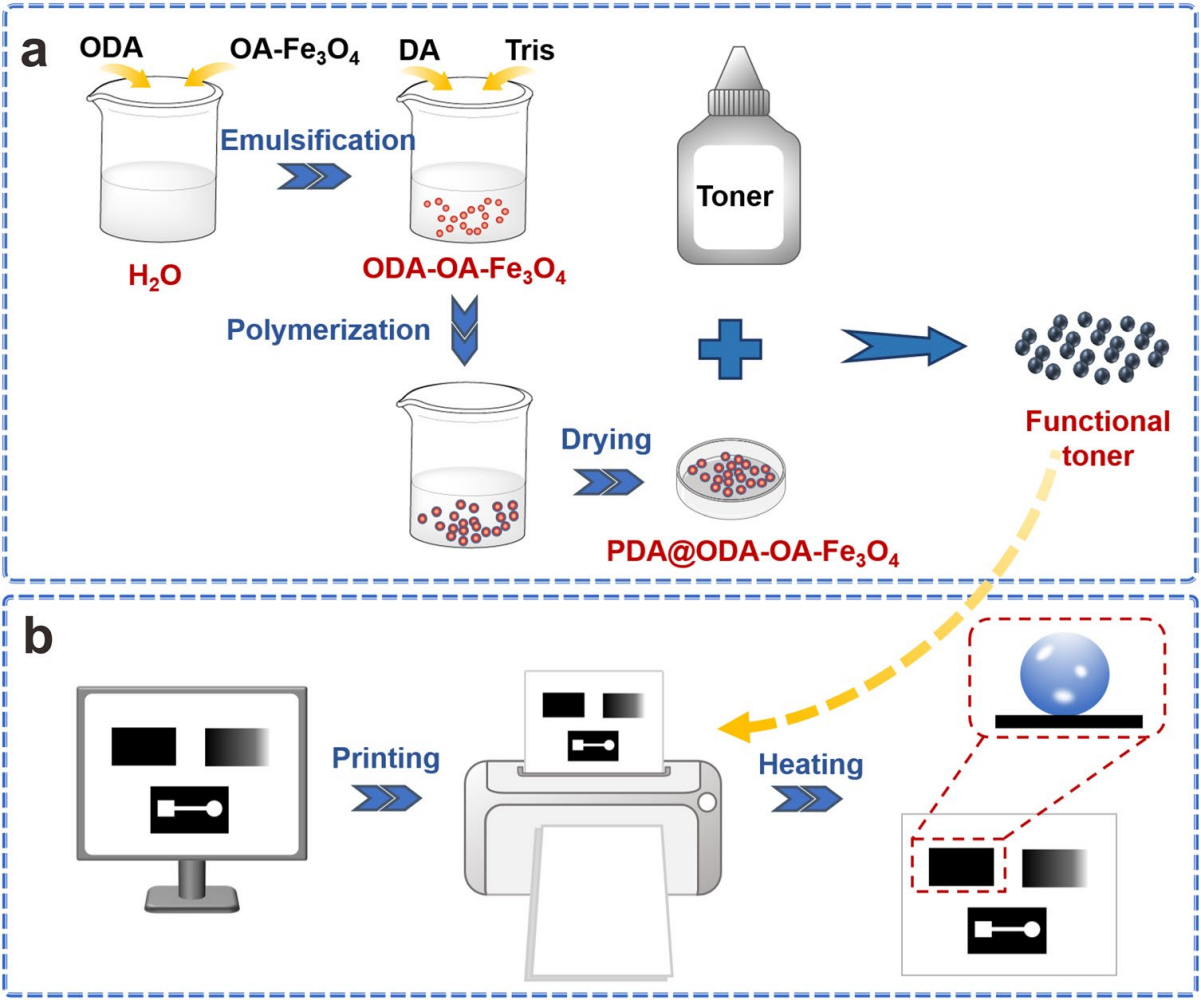
(**a**) Schematic illustration of preparation of functional toner used for laser printers, and (**b**) laser printing of paper-based devices through printing functional toner on paper substrate.

The scheme of the preparation of functional toner and the printing of paper-based devices is illustrated in Fig. 1. As described in Fig. 1a, commercial toner and PDA@ODA-OA-Fe_3_O_4_ nanocapsules were mixed uniformly, generating functional toner. The as-prepared functional toner can be used to print various paper-based superwettable patterns enabled by office laserjet printer, as demonstrated in Fig. 1b.

### Analysis and characterization of functional toner

**Figure 2:**
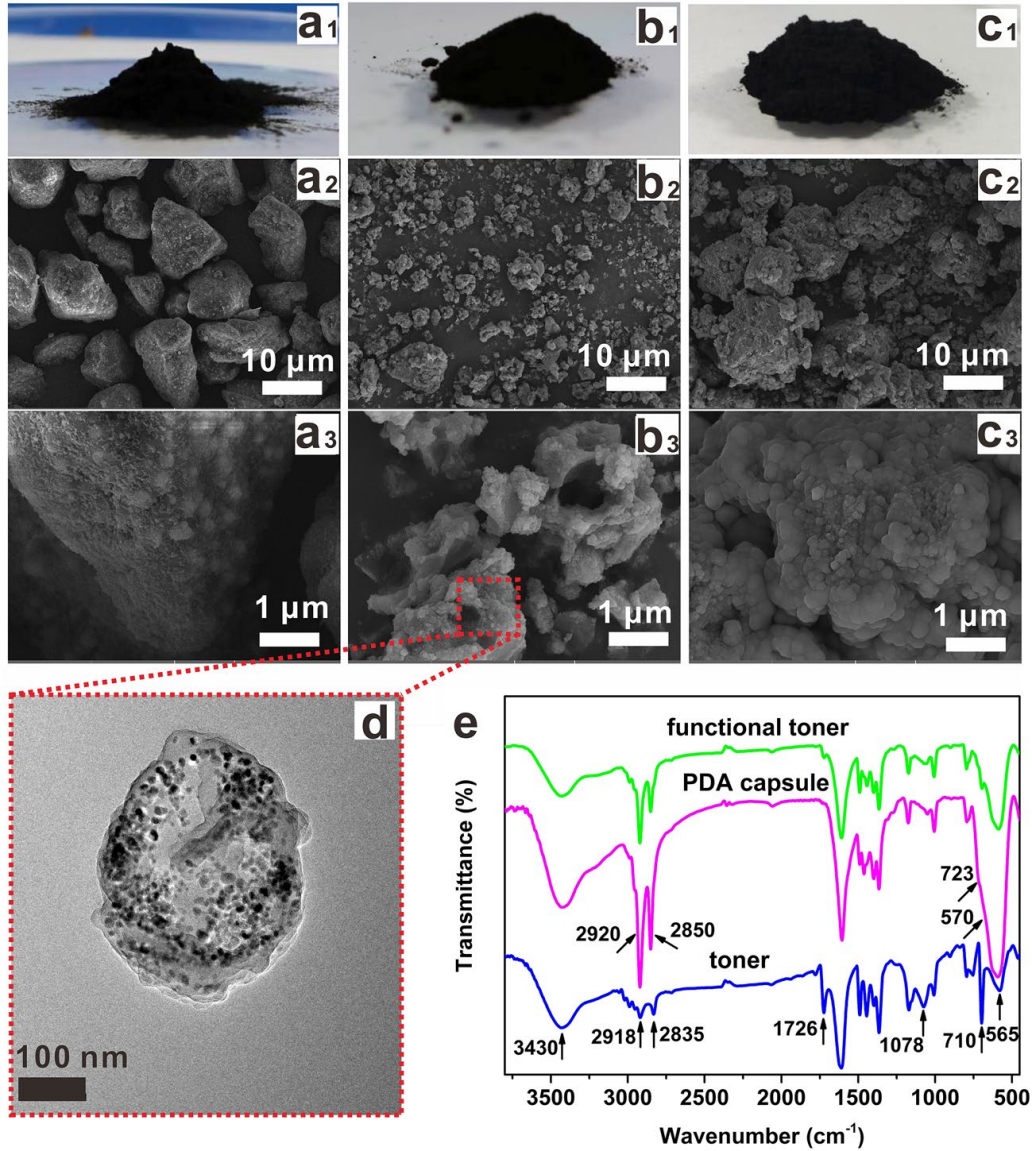
Morphologies and compositions of commercial toner, PDA@ODA-OA-Fe_3_O_4_ nanocapsules and functional toner: (**a**_**1**_–**c**_**1**_) Optical photos, (**a**_**2**_–**c**_**3**_) SEM images, (**d**) TEM image of PDA@ODA-OA-Fe_3_O_4_ nanocapsules, and (**e**) FTIR spectroscopies.

The morphologies and compositions of the commercial toner, PDA@ODA-OA-Fe_3_O_4_ nanocapsules and functional toner mixed with PDA@ODA-OA-Fe_3_O_4_ are shown in Fig. 2.

Figure 2a1–c1 show physical view of the commercial toner, PDA@ODA-OA-Fe_3_O_4_ nanocapsules and functional toner, respectively. As can be seen in Fig. 2c1, the addition of the PDA@ODA-OA-Fe_3_O_4_ nanocapsules does not affect the macroscopic state of the commercial toner, and hence the as-prepared functional toner is suitable for office laser printing technology.

The microscopic morphology of commercial toners, PDA@ODA-OA-Fe_3_O_4_ nanocapsules and functional toners were characterised using scanning electron microscopy. As can be seen in Fig. 2a2, commercial toner is particles of micron size with a uniform shape and size, and the surface of commercial toner is observed to be relatively smooth at high magnification (Fig. 2a3). Fig. 2b2,b3 show SEM images of PDA@ODA-OA-Fe_3_O_4_ nanocapsules magnified at 5000 times and magnified at 30,000 times, respectively. At low magnification, the shape of PDA@ODA-OA-Fe_3_O_4_ is large, variable, and agglomerated. At 30,000 times magnification, irregular capsules can be found to be attached together, with relatively rough surfaces and agglomeration between capsules, forming many nano cavities. The TEM image of PDA@ODA-OA-Fe_3_O_4_ nanocapsules shows in Fig. 2d that the particles are about 500 nm, the outer coating is the wall of PDA capsule, the core is the ODA and the Fe_3_O_4_ nanoparticles distributed relatively uniformly, indicating that the prepared particles are nanocapsules. As shown in Fig. 2c2, it is observed that functional toners are composed of micron blocks with rough surfaces, because PDA@ODA-OA-Fe_3_O_4_ capsules are small enough to bind to the surface of commercial toner particles. When magnified 30,000 times, as shown in Fig. 2c3, functional toner surfaces are composed of many nanometer-sized papillary spheres, resulting in very rough surfaces. Thus, when functional toner is printed on paper, the rough micro and nanostructures on the surface of functional toner form the structural basis for superhydrophobic properties.

The results of compositional analysis of commercial toner, PDA@ODA-OA-Fe_3_O_4_ nanocapsules and functional toner using infrared spectroscopy are shown in Fig. 2e. From the FTIR of the commercial toner, it can be seen that 3430 cm^−1^ is the stretching vibration peak of –OH in the polyacrylate-polystyrene copolymer in commercial toner. 2918 cm^–1^ and 2835 cm^−1^ are the C–H asymmetric stretching vibration and symmetric stretching of –CH_2_– in the polyacrylate-polystyrene copolymer and polyethylene and polypropylene as surface modifiers. 1726 cm^−1^ are the C=O stretching vibration peak in polyacrylate-polystyrene copolymers and charge modifiers. Asymmetric and symmetric stretching vibration peaks of Si–O–Si in SiO_2_ as flow agent are at 1078 cm^−1^ and 800 cm^−1^, respectively, and Fe–O stretching vibration peaks in Fe_3_O_4_ as magnetic powders is at 565 cm^−1^. As shown in FTIR of PDA@ODA-OA-Fe_3_O_4_ nanocapsules, 2920 cm^−1^ and 2850 cm^−1^ are the asymmetric and symmetric stretching vibration peak of the C–H bond of –CH_2_– respectively, and 723 cm^−1^ is the in-plane bending rocking vibration peak of the alkyl chain –(CH_2_)_n_– in PDA@ODA-OA-Fe_3_O_4_ nanocapsules. Compared to commercial toners and PDA@ODA-OA-Fe_3_O_4_ nanocapsules, FTIR of functional toner shows that it has both the characteristic peaks of commercial toner and PDA@ODA-OA-Fe_3_O_4_ nanocapsules. Hence, functional toner mixed with commercial toner and PDA@ODA-OA-Fe_3_O_4_ nanocapsules is both suitable for laser printing technology and capable of achieving superhydrophobic properties on laser printed paper-based surfaces.

### Analysis and characterization of functional toner printed paper

**Figure 3:**
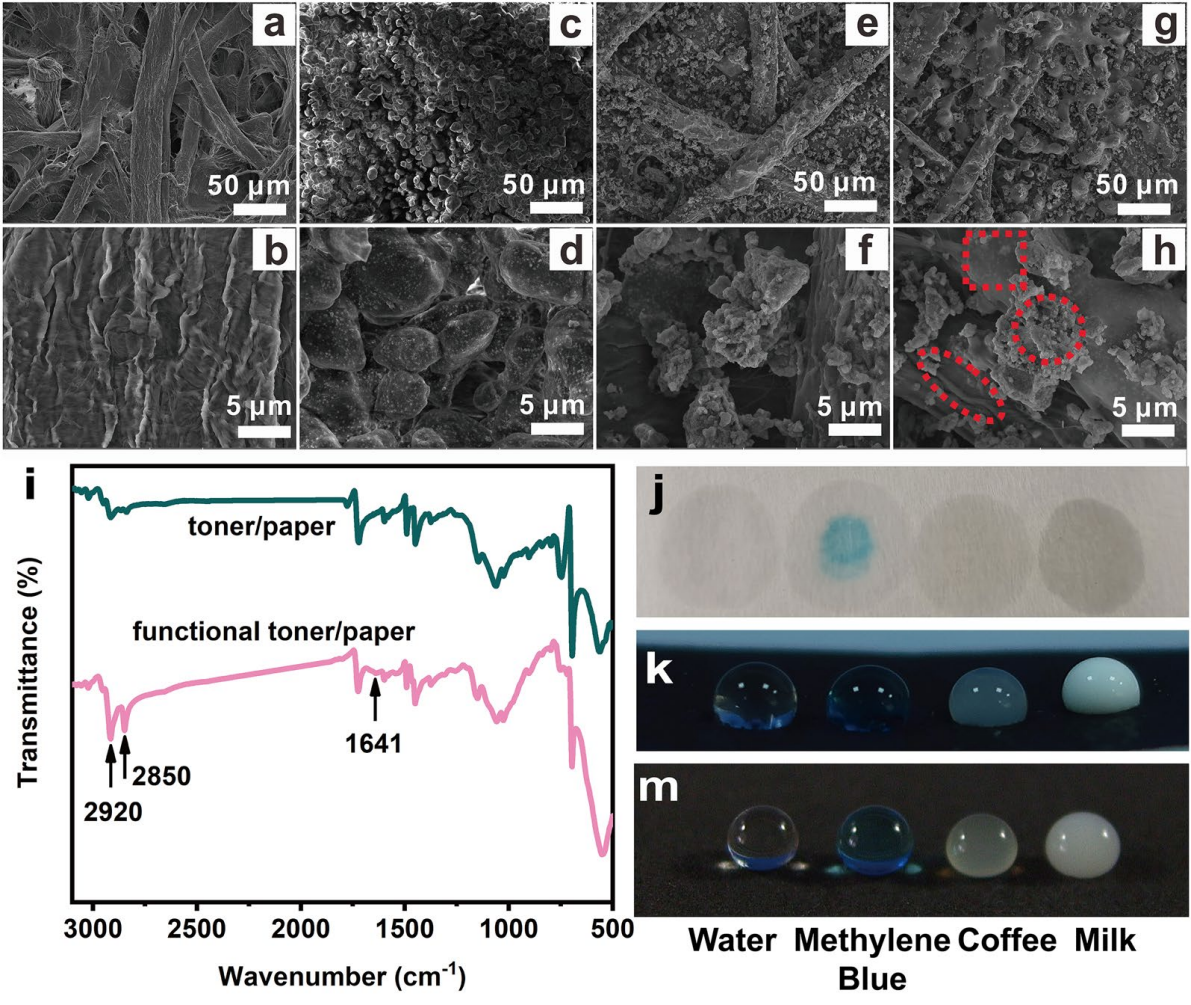
SEM images of blank, commercial toner printed, PDA@ODA-OA-Fe_3_O_4_ nanocapsules printed and functional toner printed filter paper (**a**–**h**), FTIR of commercial toner and functional toner printed filter paper, respectively (**i**), and wettability of blank, commercial toner printed and functional toner printed filter paper (**j**–**m**).

The prepared PDA@ODA-OA-Fe_3_O_4_ nanocapsules were mixed with commercial toner, where the mass of PDA@ODA-OA-Fe_3_O_4_ was 75%, to form functional toner, which was printed on the surface of filter paper to obtain paper-based superhydrophobic surface, as indicated in Fig. 3.

Figure 3a,b show the microscopic morphology of blank filter paper at 500 times and 10,000 times magnification respectively, where it is observed that the fold of cellulose on the paper surface and the relatively smooth surface of the cellulose fiber. Figure 3b clearly shows the large cavities of cellulose with strong hydrophilicity due to the presence of hydrocarbon groups. Figure 3c,d show the micromorphology of filter paper printed with commercial toner. It can be observed that the toner particle size is about 5–10 μm and distributed uniformly on the surface of cellulose fibers. Figure 3e,f present the microscopic structure of PDA@ODA-OA-Fe_3_O_4_ printed filter paper, containing many particles similar to PDA@ODA-OA-Fe_3_O_4_ nanocapsules. Figure 3g,h show the micromorphology of patterns printed with functional toner. It can be seen in Fig. 3g that papillary structure is presented on the printed surface due to the melting of ODA in the printing process, and in Fig. 3h, the nanocapsules are evenly distributed on the papilla, as shown in the red circle. In Fig. 3h**,** it can also be seen that the surface is distributed with many nanopores, allowing air to be efficiently stored and forming air cushions, thus increasing the contact area between liquid and air and improving water repellency performance.

FTIR is used to test the composition of filter paper printed with commercial toner and functional toner respectively, and the results are shown in Fig. 3i. 2920 cm^−1^ and 2850 cm^−1^ are the peaks of the asymmetric and symmetric stretching vibrations of the C–H bond of –CH_2_–, respectively. These two peaks are not clear enough in FTIR of filter paper printed with commercial toner, whereas they are very obvious in FTIR of functional toner printed paper due to the presence of alkyl chains in ODA encapsulated in PDA@ODA-OA-Fe_3_O_4_ nanocapsules. The peak at 1641 cm^−1^ is assigned to the C=N stretching vibrations of Schiff base reaction product between PDA and ODA^37^. The FTIR results show that the presence of the PDA@ODA-OA-Fe_3_O_4_ nanocapsules introduces alkyl chains for functional toner, effectively reducing the surface tension in the printed area and achieving good superhydrophobic properties.

Based on the rough structure of micro nano-scales and alkyl chains with low surface-energy, functional toner-printed paper has good superhydrophobic properties. Deionized water, methylblue, coffee and milk drops were applied to qualitatively prove the wettability. As can be seen from Fig. 3j, each of the four droplets penetrated into the blank filter paper and left liquid stains on the surface of blank filter paper. As shown in Fig. 3k, the four droplets remain in a hemispherical shape on the surface of filter paper printed with commercial toner, but the hemispherical droplets can only be held for 10 s and then absorbed by the printed filter paper (Movie S1–S4). The contact angles of deionized water, methylene blue solution, coffee and milk were 121°, 123°, 126°, and 98° respectively, as shown in Table 1. In contrast, on the surface of the filter paper printed with functional toner, the four droplets remained in the shape of spheres, and the contact angles of deionized water, methylene blue solution, coffee and milk are 153°, 155°, 150°, and 148° respectively, as shown in Fig. 3m and Table 1. Moreover, the droplets are stable on the filter paper surface printed with functional toner and cannot be absorbed (Movie S5–S8). All the results indicate that the paper surface printed with functional toner achieves excellent superhydrophobic properties.

**Table 1:** CA of various liquid droplets on paper-based samples.

Liquids	Water	Methylene blue solution	Coffee	Milk
Blank paper	0°	0°	0°	0°
Commercial toner printed paper	121°	123°	126°	98°
Functional toner printed paper	153°	155°	150°	148°

### Optimization of the content of PDA@ODA-OA-Fe_3_O_4_ nanocapsules in functional toner

**Figure 4:**
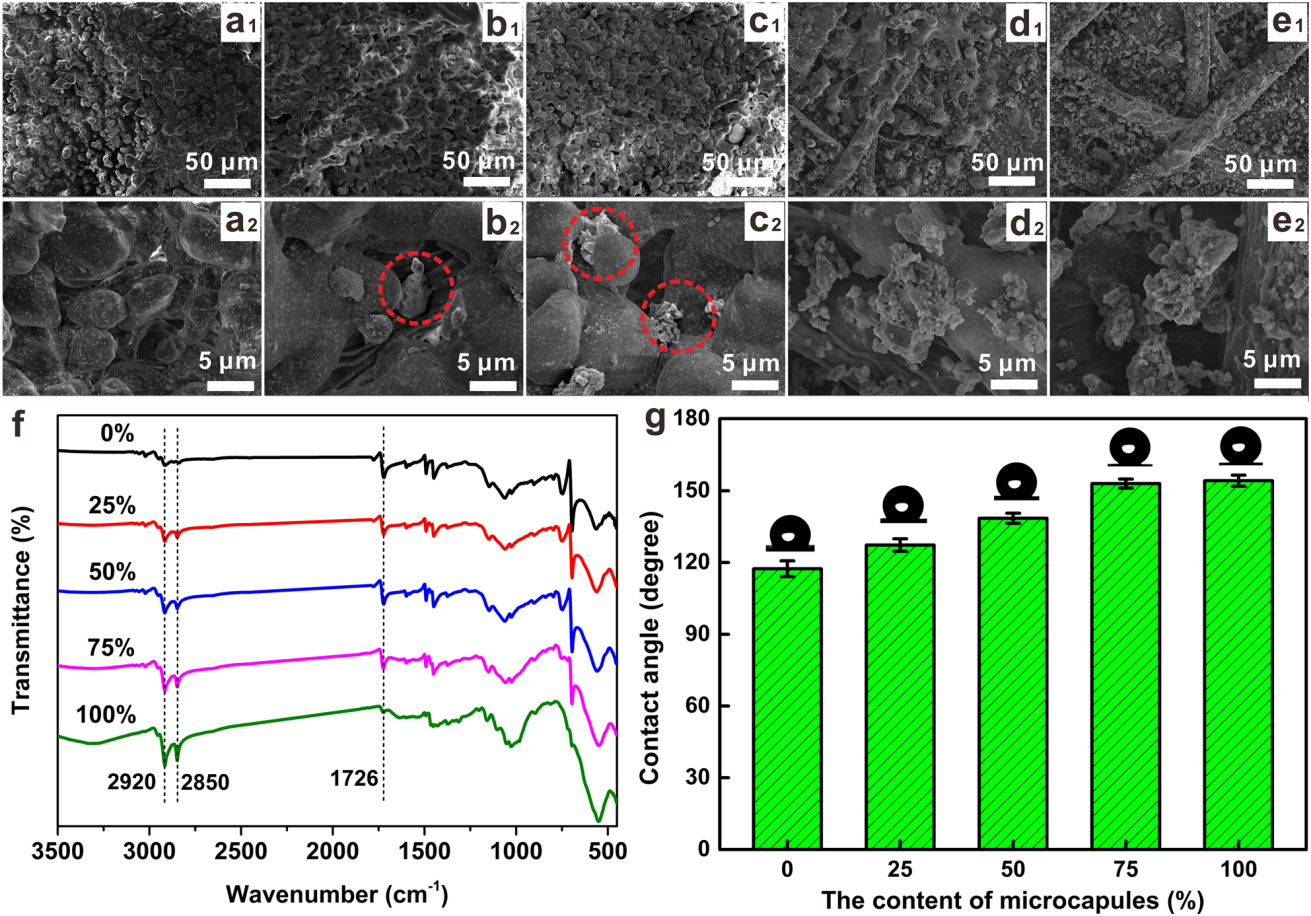
Effect of different contents of PDA@ODA-OA-Fe_3_O_4_ nanocapsules on morphologies, compositions and superwettability of printed filter: SEM images of filter printed with functional toner including 0% (**a**_**1**_–**a**_**2**_), 25% (**b**_**1**_–**b**_**2**_), 50% (**c**_**1**_–**c**_**2**_), 75% (**d**_**1**_–**d**_**2**_), and 100% (**e**_**1**_–**e**_**2**_) PDA@ODA-OA-Fe_3_O_4_ nanocapsules, respectively, FTIR (**f**) and superwettability (**g**) of filter printed with different functional toner.

In functional toner, the mixture weight ratio of commercial toners and PDA@ODA-OA-Fe_3_O_4_ nanocapsules is different, resulting in different wettability of the printed surface. In order to optimize the content of PDA@ODA-OA-Fe_3_O_4_ nanocapsules, it is investigated the effect of different contents of nanocapsules on the wetting performance of printed paper, in which the microcapsule contents are 0 wt%, 25 wt%, 50 wt%, 75 wt% and 100 wt%, as shown in Fig. 4.

As shown in Fig. 4a–e, the SEM image was observed at 500 and 10,000 times to compare the morphology of the printed surfaces using functional toners with different dosage of nanocapsules. The surface printed with functional toner with 0 wt% nanocapsules, i.e. pure toner, is covered by micron-sized toner particles, and the toner surface is relatively smooth, as shown in Fig. 4a1,a2. Filter paper surfaces printed with functional toners of 25 and 50 wt% nanocapsules are covered with large amounts of commercial toners and very small amounts of PDA@ODA-OA-Fe_3_O_4_ nanocapsules, which exposes large amounts of smooth surfaces of commercial toner, but only adhere very small amounts of nanocapsules, as shown in the red circles in Fig. 4b2,c2. When the microcapsule content is 75 wt%, as shown in Fig. 4d1,d2, no obvious toner particles can be observed on the surface because PDA@ODA-OA-Fe_3_O_4_ nanocapsules are completely attached to the toner surface, and PDA@ODA-OA-Fe_3_O_4_ nanocapsules are combined to form many nanoscale cavities. Similarly, when the capsule content is 100 wt%, the printed surface is full of PDA@ODA-OA-Fe_3_O_4_ nanocapsules with a very rough surface observed from Fig. 4e1,e2.

FTIR of paper-based surface printed with functional toners with different microcapsule contents were studied and the results are shown in Fig. 4f. Two peaks at 2920 cm^−1^ and 2850 cm^−1^ attributed to C–H asymmetry and symmetric stretching vibrations become stronger with increasing microcapsule content in functional toners, which indicates that the increase of microcapsule content can introduce more ODA, thus further enhancing the superhydrophobic properties of the printed surface.

According to the above-mentioned analysis, the rough surfaces consisting of functional toner form a structural basis for the construction of superhydrophobic surfaces, while the hydrophobic ODA in the PDA@ODA-OA-Fe_3_O_4_ nanocapsules forms a compositional basis for the construction of superhydrophobic surfaces. Thus, the printed surface has a superhydrophobic property if the nanocapsule content in functional toner is 75 wt%, and the WCA of printed surface is 153.0°, as shown in Fig. 4g. Consequently, the functional toner with a nanocapsule content of 75 wt% has the best superhydrophobic property and printing performance.

### Superwettable patterns printed with functional toner

In order to demonstrate the printing performance of the prepared functional toner, patterns of different shapes and different grey levels were designed on the computer, and all the designed patterns printed with functional toner are shown in Supplementary Fig. S2. It can be seen that various patterns can be printed based on the controllable and convenient features of laser printing technology.

**Figure 5:**
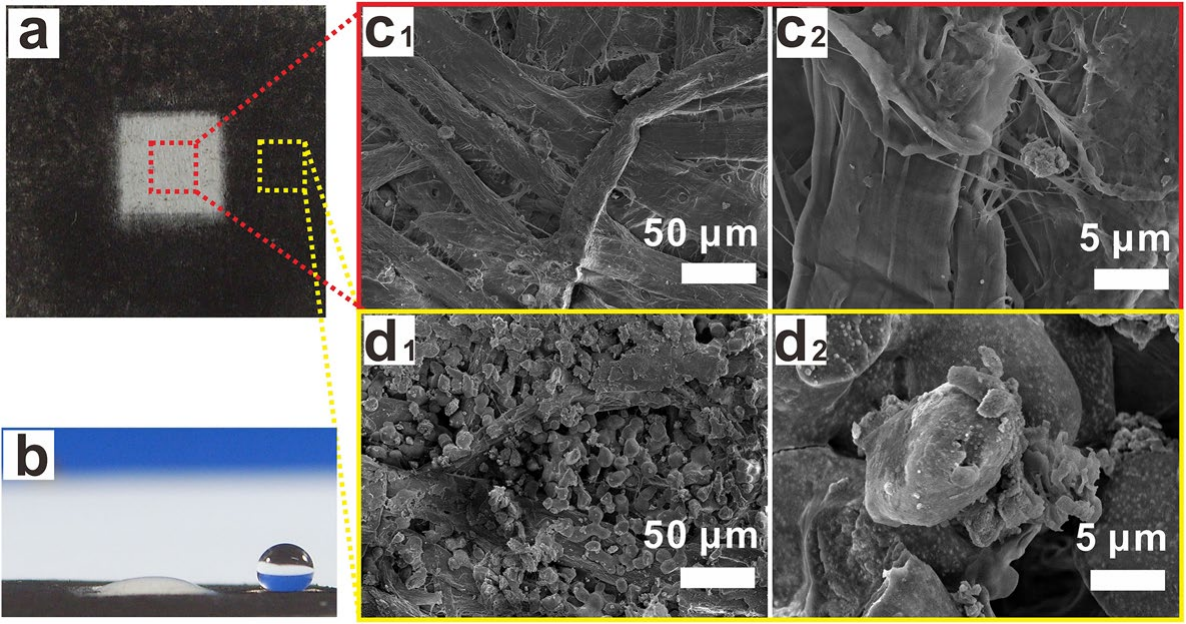
Pattern printed with functional toner on filter paper: (**a**) Optical photo of printed pattern, (**b**) Wettability of printed pattern, (**c**_**1**_–**c**_**2**_) SEM micrographs of the blank area of printed pattern, and (**d**_**1**_–**d**_**2**_) SEM micrographs of the black area of printed pattern.

One of the patterns is selected to test the surface wettability and observe the surface microscopic structure, as shown in Fig. 5. The optical photo of the printed pattern is shown in Fig. 5a, which consists of an external printed square and an internal unprinted square. As can be seen from Fig. 5b, the water droplet is spherical and the WCA is 152.5° in the printed area while water droplets penetrated into the filter paper in the unprinted aera. The surface micromorphology can be seen from Fig. 5c1,c2 that in the non-printed area there is only cellulose fiber of the filter paper, while in printed paper, as shown in Fig. 5d1,d2, nanocapsules are attached to the surface of commercial toners, covering the cellulose surface, and forming rough nanopores on the surface of the filter paper. The results demonstrate that functional toner is capable of printing patterned surface with controllable wettability.

**Figure 6:**
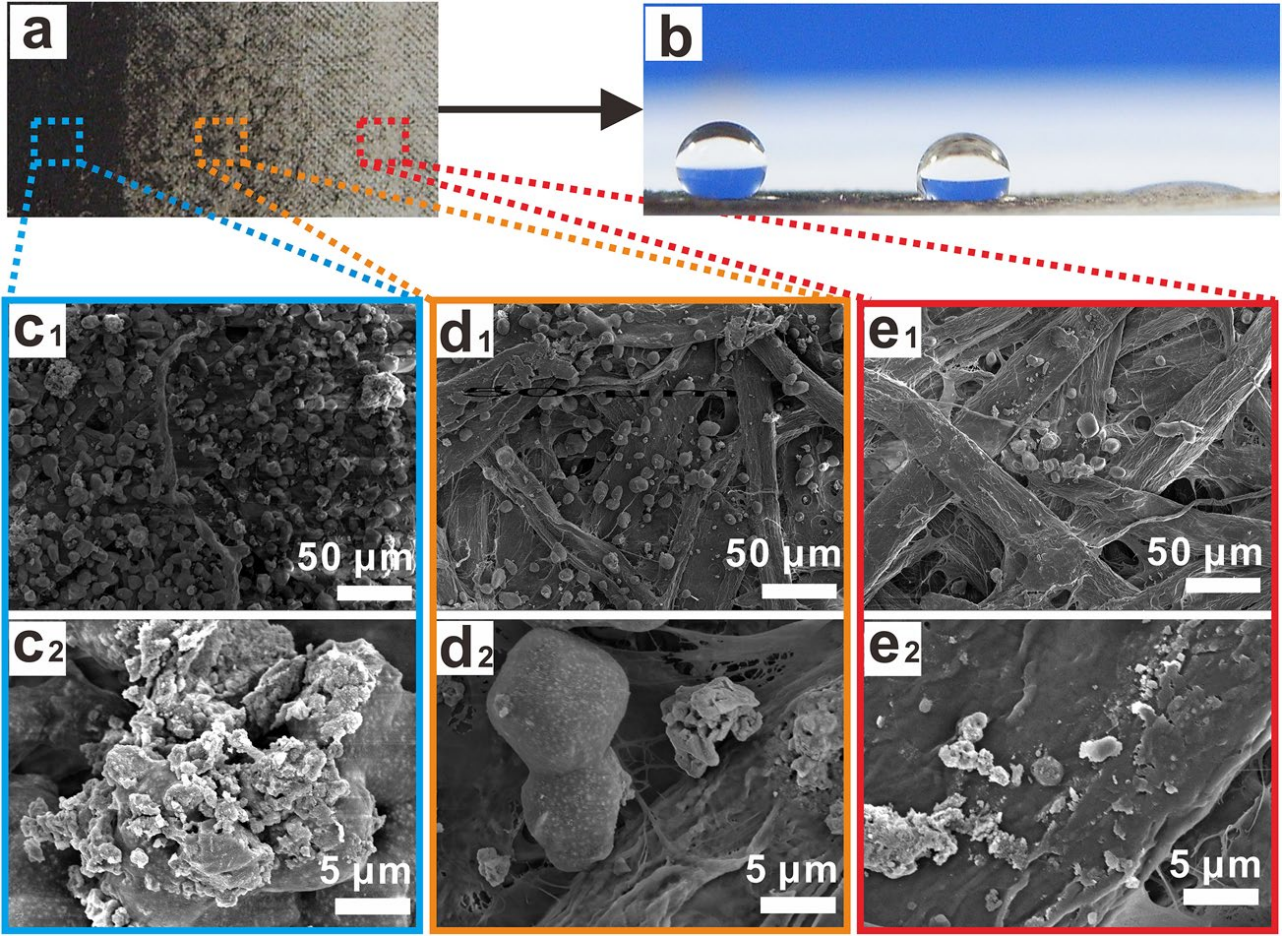
Gradient pattern printed with functional toner on filter paper: (**a**) Optical photo of printed gradient pattern, (**b**) Wettability of printed gradient pattern, SEM micrographs of the darkest area (**c**_**1**_–**c**_**2**_), the darker area (**d**_**1**_–**d**_**2**_) and the lightest area (**e**_**1**_–**e**_**2**_) of printed gradient pattern.

Figure 6 shows gradient pattern in different grey levels of printing with functional toners, and the color of the paper surface gradually lightens from left to right (Fig. 6a). Its wettability also shows gradient change from left to right (Fig. 6b). As can be seen from Fig. 6b, the water droplet is spherical and the WCA is 151° in the area marked by blue line, showing superhydrophobic property, and the water droplet is hemispherical and the WCA is 101° in the area marked by orange line, showing hydrophobicity, while the area marked by red line has WCA of 6° and exhibits hydrophilic property. The micromorphology in the area marked with blue line (Fig. 6c1,c2) indicates that the filter paper surface of is completely covered with uniform functional toner. In the orange-lined area, the SEM image shows that some fibers of filter paper are exposed to the surface, with only a small amount of functional toner on it (Fig. 6d1,d2). In the red line area, only a small number of functional toners are printed on the surface of the filter paper (Fig. 6e1,e2), showing strong hydrophilicity in the light area. The results demonstrate that the prepared functional toners can print patterns of different grey levels, thus enabling the gradation of superhydrophobic property on the printed paper.

### Durability and stability of superwettable patterns printed with functional toner

**Figure 7:**
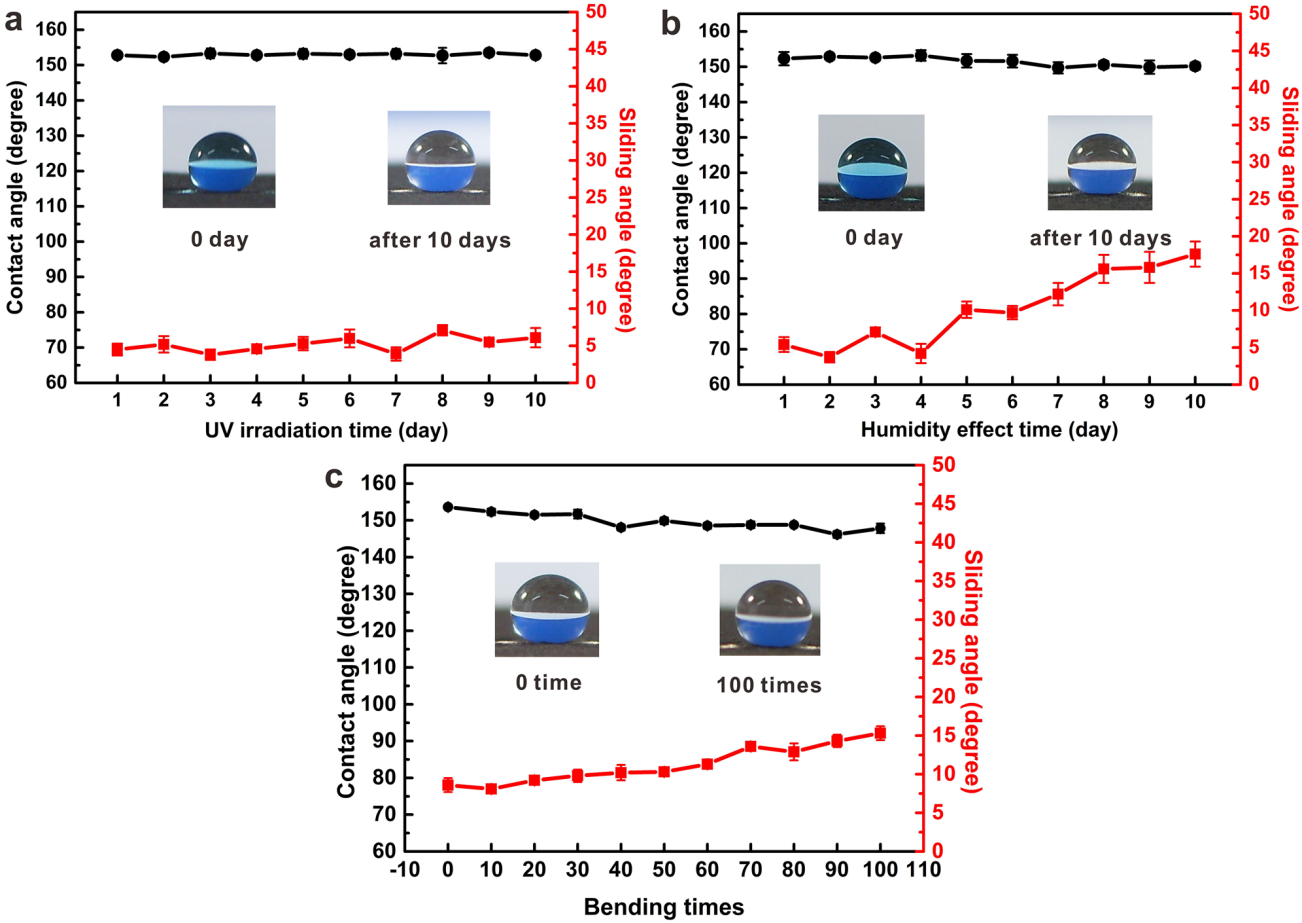
Evolution of WCA and SA at the superhydrophobic surfaces printed with functional toner upon diversified circumstances: (**a**) UV irradiation for 10 days, (**b**) placed in a constant humidity chamber at 95% humidity for 10 days, and (**c**) resistance to bending upon 100 times.

In order to investigate the durability and stability of the paper-based superhydrophobic surface printed with functional toner, a series of exploration tests were conducted and the evolution of WCA and SA on the printed surface with different circumstances were examined, as shown in Fig. 7.

The UV resistance is tested by investigating the WCA and SA evolution when irradiating the printed surface using functional toner under UV light with a wavelength of 365 nm, and the obtained result is presented in Fig. 7a. As can be seen from the graph, after 10 days of exposure to UV light, the WCA on the printed filter paper remained at about 150° and the SA also remained within 10° with no significant change. It indicates that the UV light did not damage the superhydrophobicity, and the surface of filter paper printed with functional toner has excellent durability and stability under UV irradiation.

Figure 7b shows the change of WCA and SA when the printed filter paper has been placed in the constant temperature and humidity chamber with 95% humidity and constant temperature of 20 °C for 10 days. With the extension of the placement time, the WCA on the printed surface tends to decrease gently and is not less than 145°. The SA is less than 10° within 7 days, and rises slightly after 7 days, but the change is not significant and does not exceed 15°. It indicates that the surface of filter paper printed with functional toner has good moisture resistance.

A bending examination is carried out to test the durability and stability of the surface printed with the as-prepared functional toner, as shown in Fig. 7c. The WCA is almost no change in spite of repeated bending 100 cycles, and the SA is slightly elevated but remains within 15°. It manifests that the printed surface using functional toner demonstrates excellent durability and flexibility due to the firm bonding of functional toner on the filter paper.

All results above prove that the surface of filter paper printed with as-prepared functional toner exhibit excellent durability and stability, and which indicates that the printed paper-based surface can effectively resist the damage for paper. This is of great significance for using the functional toner to print paper-based devices.

### Proof-of-concept of ions testing using functional toner printed paper-based devices

**Figure 8:**
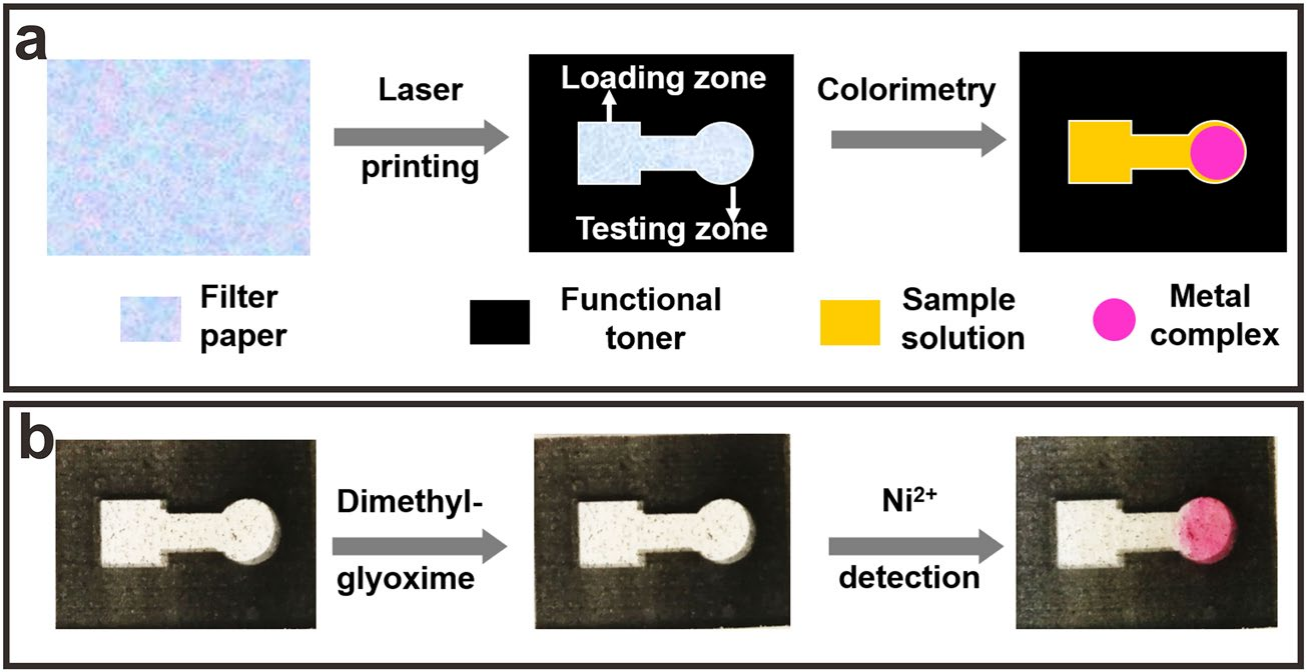
Demonstration of using the paper-based devices printed with functional toner for point-of-care testing of metal ions: (**a**) scheme of fabrication process, and (**b**) point-of-care testing of nickel ions.

In order to demonstrate the functionality of paper-based devices printed with functional toner for point-of-care testing, a kind of μPADs is designed and printed with functional toner for the colorimetric detection of metal ions, as shown in Fig. 8.

The μPADs is designed using Microsoft PowerPoint 2016 software and is shown in Fig. 8a, where the square area is the sample loading zone and the circular area is the testing zone, and the sample loading zone and testing zone connected by a rectangular channel. All the loading zone, detection zone and connection channel are designed as non-printing aera and would be hydrophilic, while the surrounding aera is designed as printing aera and is superhydrophobic due to the coating of functional toner. For detection, the metal ion indicator is first added to the detection zone and dried, and then the sample solution is added dropwise to the loading zone. The liquid to be measured moves through the hydrophilic channel to the detection area and undergoes a distinct color change, thus enabling colourimetric detection of metal ions.

The paper-based microfluidic device is printed for Ni^2+^ detection as shown in Fig. 8b. As reported by Zhang et al.^17^, dimethylglyoxime is chosen as the chromogenic reagent and added in the detection zone and then dried at room temperature, and then 100 μL aqueous solution containing Ni^2+^ is added dropwise to the sample zone. The sample solution diffused through the hydrophilic channel to the detection zone, resulting in a deep wine color within 30 s. The result indicates that the μPADs printed with functional toner can be used to the fast and qualitative detection of Ni^2+^. Based on a similar principle, other metal ions also can be detected in this way, as shown in the Supplementary Fig. S3. This demonstration of using functional toner provides new opportunities for colorimetric PADs with mass production as well as do-it-yourself.

**Figure 9:**
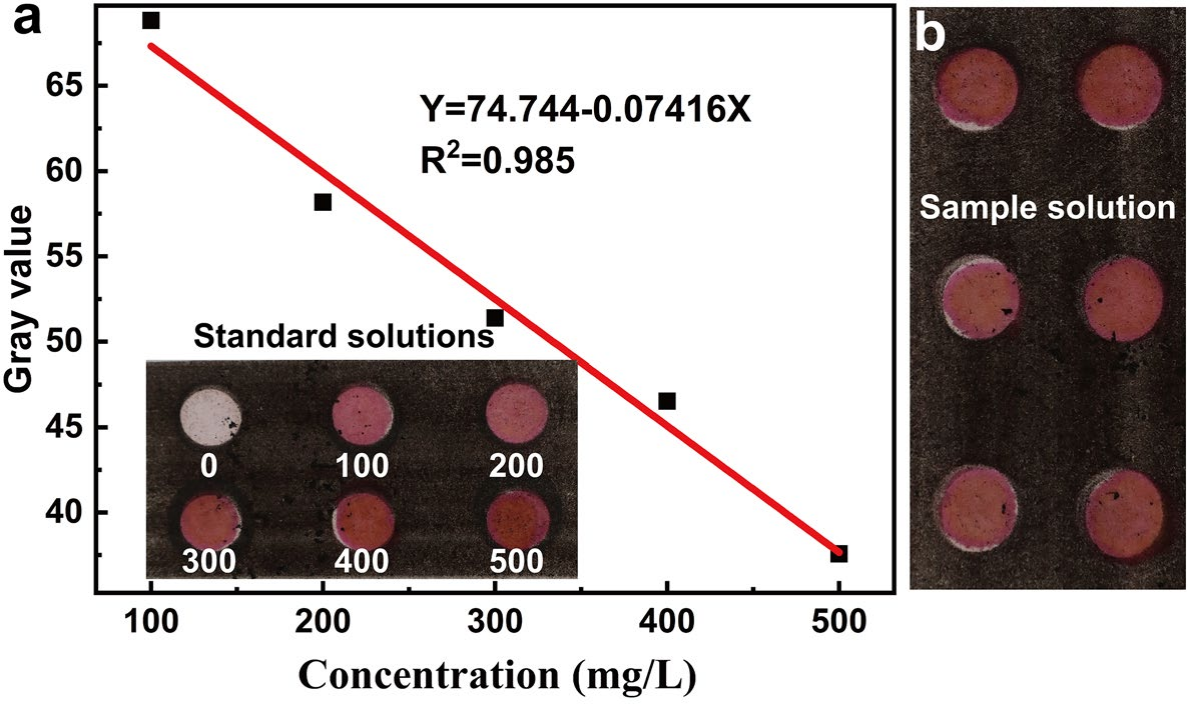
Quantitative of nitrite: (**a**) the change of gray value with the concentration of nitrite and the linear calibration equation obtained by fitting the gray value of standard solutions; (**b**) the variation in color of sample solution.

The quantitative detection of nitrite ions is shown in Fig. 9. The Griess reagent prepared in ethanol was dropped into the detection zones, and it was confined by the round hydrophobic barrier. The nitrite standard solutions were added respectively into the round patterns and the pink color developed^26^, as demonstated in the insert of Fig. 9a. The colormetric gray values were obtained using ImageJ sofeware Fig. 9a displays the change of gray value with the concentration of nitrite and the linear calibration equation obtained by fitting the gray value of standard solutions. The sample of nitrite solution was dropped into the detection zone of the printed device and the variation in color was clearly observed (Fig. 9b), and its gray value was measured as 52.24. Its concentration was calculated as 303.45 mg/L using the fitted standard equation, which is not much different from the prepared concentration (300 mg/L). The results indicate that the paper-based device printed with functional toner can be used for quantitative detection of various ions.

## Conclusions

In this work, we have developed a kind of functional toner for office laser printers consisting of a mixture of PDA@ODA-OA-Fe_3_O_4_ nanocapsules and commercial toners, which can be used for printing superhydrophobic paper-based surfaces with CA greater than 150°. The effect of PDA@ODA-OA-Fe_3_O_4_ nanocapsules content in the functional toner on the wettability of printed surfaces has been studied. Increasing the content of PDA@ODA-OA-Fe_3_O_4_ nanocapsules resulted in better hydrophobicity with greater contact angles, and the result shows that the best content of PDA@ODA-OA-Fe_3_O_4_ nanocapsules is 75 wt%. In addition, functional toner can be used to print a variety of patterns designed using computer software, and the morphologies of the patterns prove that functional toner is clearly present in the printed area but not in the unprinted area, which indicates that the as-prepared functional toner has good print performance. Moreover, the durability under UV light and moisture and the mechanical stability of the printed paper-based surfaces with functional toner have been investigated and the printed surfaces exhibit excellent durability and stability. More importantly, the functionality of the paper-based devices printed using the as-prepared functional toner has been demonstrated successfully through colorimetric test for metal and nitrite ions. Our study provides a new strategy for converting the regular office laser printer into a high throughput fabrication platform of paper-based devices such as point-of-care sensing, diagnostics and paper electronics through the design and preparation of functional toner.

### Supplementary Information

Supplementary Figures.

Supplementary Video S1.

Supplementary Video S2.

Supplementary Video S3.

Supplementary Video S4.

Supplementary Video S5.

Supplementary Video S6.

Supplementary Video S7.

Supplementary Video S8.

## Data Availability

All data generated or analyzed during this study are included in this published article.
