# Supplementary material for: Functional toner for office laser printer and its application for printing of paper-based superwettable patterns and devices
Source: Sci Rep. 2023 Aug 3;13:12592. doi: 10.1038/s41598-023-39729-8 (PMC10400629; doi:10.1038/s41598-023-39729-8)
Supplement: Supplementary file 1 — Supplementary Figures. [file 41598_2023_39729_MOESM1_ESM.docx]

Supplementary Information

Functional Toner for Office Laser Printer and Its Application for Printing of Paper-based Superwettable Patterns and Devices

Yanhua Liu* • Xingfei Liu • Juanning Chen • Zhuanli Zhang • Libang Feng*

School of Materials Science and Engineering, Lanzhou Jiaotong University, Lanzhou 730070, China


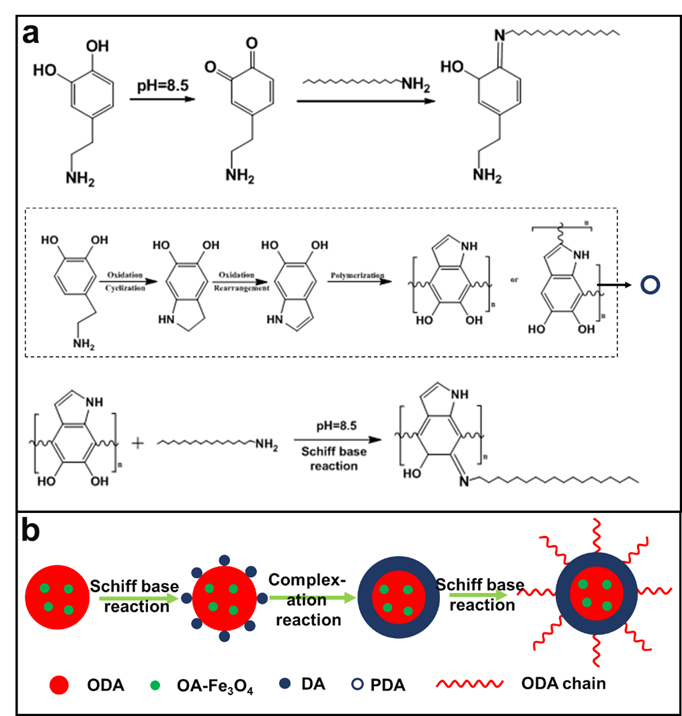


Fig. S1. Formation mechanism of PDA@ODA-OA-Fe_3_O_4_ nanocapsules


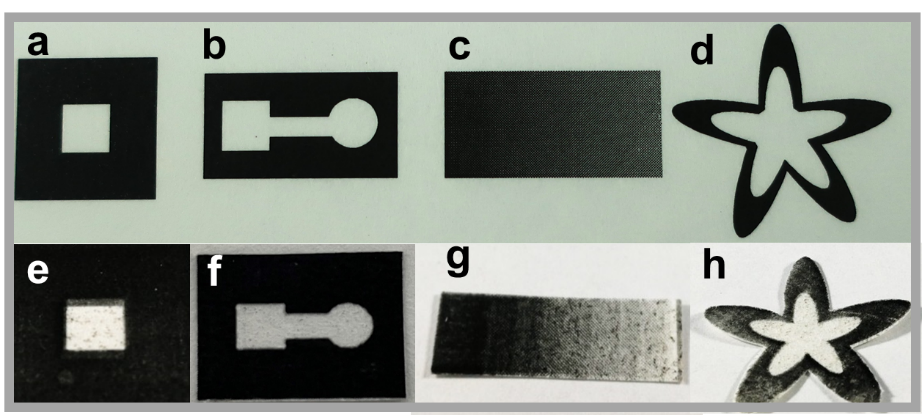


Fig. S2. Various patterns printed using commercial toner (a-d) and functional toner (e-h)


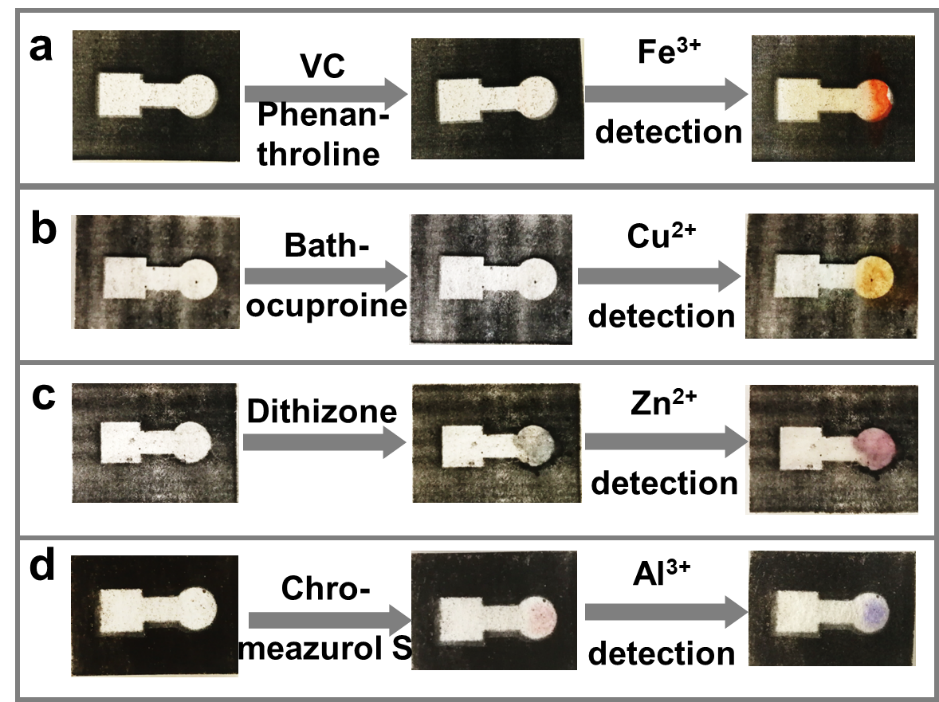


Fig. S3. paper-based devices printed with functional toner for point-of-care testing of metal ions: (a) Fe^3+^, (b) Cu^2+^, (c) Zn^2+^ and (d) Al^3+^
